# 糖皮质激素对免疫检查点抑制剂疗效影响的研究进展

**DOI:** 10.3779/j.issn.1009-3419.2019.12.09

**Published:** 2019-12-20

**Affiliations:** 1 325035 温州，温州医科大学 Wenzhou Medical University, Wenzhou 325035, China; 2 310022 杭州，浙江省肿瘤医院胸部肿瘤内科 Department of Thoracic Oncology, Zhejiang Cancer Hospital, Hangzhou 310022, China

**Keywords:** 检查点抑制剂, 糖皮质激素, 免疫治疗, Checkpoint inhibitors, Steroids, Immunotherapy

## Abstract

免疫检查点抑制剂（immune checkpoint inhibitors, ICIs）包括程序性死亡受体-1/配体-1单抗和细胞毒性T淋巴细胞相关抗原-4单抗等，因其良好的抗肿瘤活性，目前已被批准用于多种晚期恶性肿瘤的治疗。对于接受ICIs治疗的患者，由于肿瘤的部分并发症、免疫治疗相关不良反应以及免疫联合化疗前预处理等，通常会在ICIs治疗过程中使用糖皮质激素进行干预及处理。但超生理剂量的糖皮质激素会对机体产生一定的免疫抑制作用，甚至可能影响ICIs的疗效。因而临床上对于ICIs治疗患者激素的使用存在一定争论。本文就糖皮质激素对ICIs疗效影响的研究进展进行综述。

## Abstract (en)

Immune checkpoint inhibitors (ICIs), including programmed death receptor-1 (PD-1)/ligand (PD-L1) and cytotoxic T-lymphocyte-associated antigen-4 (CTLA-4) monoclonal antibody, have been approved for the treatment of multiple advanced malignant tumors, on account of its ideal antitumor activity. For patients receiving ICIs treatment, glucocorticoid intervention and treatment are often applied because of some complications associated with tumor, immunotherapy-related adverse reactions, and pretreatment before immunotherapy combined with chemotherapy. However, excessive physiological dose of glucocorticoid has a certain immunosuppressive effect, which may even affect the curative efficacy of ICIs. Thus, there are controversies about the use of steroids in patients receiving ICIs treatment. In this article, we reviewed the progress of steroids effect on the efficacy of ICIs.

近年来，以细胞毒性T淋巴细胞相关抗原-4（cytotoxic T lymphocyte-associated antigen-4, CTLA-4）单抗和程序性死亡受体1（programmed death 1, PD-1）及其配体（programmed death ligand 1, PD-L1）单抗为代表的免疫检查点抑制剂（immune checkpoint inhibitors, ICIs），已被应用于多种恶性肿瘤的治疗^[1-4]^，特别在肺癌领域，多个PD-1/PD-L1单抗已被纳入了非小细胞肺癌（non-small cell lung cancer, NSCLC）的二线及一线治疗^[5-9]^。糖皮质激素（glucocorticoid, GC）是一类重要的类固醇激素。外源性糖皮质激素包括泼尼松、地塞米松、甲基强的松龙等，通常对免疫系统具有一定的负性调控作用^[10]^。在ICIs治疗过程中，肿瘤患者常面临应用外源性激素的情况，如处理脑转移以减轻预防脑水肿^[11]^，改善纳差、呼吸困难、乏力等肿瘤伴随并发症^[12, 13]^，治疗中-重度的免疫治疗相关不良反应（immune-related adverse events, irAEs）^[14]^以及进行免疫联合化疗前预处理以减少胃肠道反应和过敏事件^[15]^等。对于GC是否会影响ICIs抗肿瘤疗效这一问题，目前有一些研究进行了探索及研究，本文对此问题进行系统综述。

## 1 糖皮质激素与免疫治疗

CTLA-4是初始T细胞活化阶段被诱导表达的检查点分子，对B7（CD80/CD86）分子具有很高的亲和力，可与CD28分子竞争B7而阻断共刺激通路，弱化T细胞受体（T-cell receptor, TCR）信号，进而抑制T细胞的活化。CTLA-4亦可通过上调调节性T细胞（T regulatory, Treg）活性及下调抗原提呈细胞B7分子的表达等途径，发挥免疫抑制效应^[16, 17]^。PD-1/PD-L1是另一对重要的免疫检查点分子，其相互作用的信号可通过抑制T细胞增殖活化^[18]^，诱导Treg分化并维持Treg功能^[19]^及促进T细胞凋亡^[20]^等途径，进而负性调控T细胞活性。ICIs通过阻断上述两类主要的免疫检查点，解除它们的“免疫刹车”作用，从而激活及强化机体的免疫系统（主要为T细胞系统），发挥抗肿瘤能力。GC对T细胞系统存在多机制的抑制效应。研究表明，GC可通过诱导T细胞的凋亡^[10]^、抑制IL-2介导的效应T细胞的增殖活化^[21]^、促进CTLA-4、PD-1表达^[22]^及增加Treg的数目比例^[23, 24]^等多种机制，对T细胞活性产生抑制作用。因此，基于上述理论，有研究者^[25-27]^认为：在ICIs治疗期间使用GC，可能会部分抑制ICIs的抗肿瘤效能，而这种猜测也确实在一些相关临床研究中得到证实。

接受ICIs治疗的肿瘤患者在其疾病的不同阶段，基于不同的治疗目的，可能存在多种GC使用指征。例如，在ICIs治疗前后，激素常用于免疫联合化疗前预处理及用于改善颅内高压、疲劳、呼吸困难等多种肿瘤伴随并发症^[11-13, 15]^；在ICIs治疗之后，激素常用于较严重的irAEs的干预处理等^[14]^。当前，有研究^[28]^报道，在ICIs治疗早期使用GC（主要指启动ICIs治疗30 d内）对ICIs疗效存在较明显的抑制作用，但同时也有个别研究得出了不同的结论。而另有部分研究^[29-33]^发现，GC用于irAEs管理并未显著影响患者的生存和疾病缓解。考虑到irAEs通常出现在启动ICIs治疗一段时间之后，上述不同的现象提示我们：在ICIs治疗的不同阶段使用GC，其对患者疗效的影响可能存在差异。

## 2 GC应用于ICIs治疗早期

### 2.1 早期GC持续暴露对ICIs疗效的影响

Kathryn等^[25]^开展了一项评估基线GC应用对PD-1/PD-L1单抗疗效影响的回顾性研究。研究将启动PD-1/PD-L1单抗治疗首日起，接受不低于10 mg/d泼尼松当量激素定义为基线应用GC，共纳入了2个中心的640例非小细胞肺癌（non-small cell lung cancer, NSCLC）患者。640例研究对象中90例存在基线GC治疗，其主要的激素适应证为呼吸困难、疲劳以及脑转移。分析显示：纪念斯隆凯特林癌症中心（Memorial Sloan Kettering Cancer Center, MSKCC）的455例患者中，基线GC组的客观缓解率（objective response rate, ORR）显著低于无激素组（6% *vs* 19%, *P*=0.02），无进展生存期（progression-free survival, PFS）及总生存期（overall survival, OS）也均较无激素组显著缩短（中位PFS：1.9个月*vs* 2.6个月，HR=1.7，*P*=0.001；中位OS：5.4个月*vs* 12.1个月，HR=2.1，*P* < 0.001）。在古斯塔夫•鲁西癌症中心（*n*=185），基线GC组的ORR尽管下降并不明显（8% *vs* 18%, *P*=0.2），但PFS及OS均显著缩短（中位PFS：1.7个月*vs* 1.8个月，HR=1.5，*P* < 0.001；中位OS：3.3个月*vs* 9.4个月，HR=2.0，*P* < 0.001）。合并2个中心的结果后显示：基线GC使用显著降低PD-1/PD-L1单抗治疗患者的ORR（*P*=0.005），缩短了患者的PFS及OS（PFS/OS: *P* < 0.001）。考虑到两组患者体能状况、脑转移史基线不均衡，研究者进行了多因素分析，结果仍提示：基线激素暴露为PD-1/PD-L1单抗治疗患者PFS及OS预后不良的独立危险因素（PFS: HR=1.31, *P*=0.03; OS: HR=1.66, *P* < 0.001）。另外，该研究深入探讨了PD-1/PD-L1单抗治疗前后不同时期GC暴露对疗效影响的差异。分析的MSKCC（*n*=455）队列中，66例患者在PD-1/PD-L1单抗治疗前30 d有GC暴露，53例患者基线使用GC，另有324例无GC治疗（包括 < 10 mg/d泼尼松当量）。结果显示：基线激素组的ORR较治疗前激素组降低（6% *vs* 8%），无激素组的ORR最高，达21%。三组的PFS及OS也存在同上对比关系，差异有统计学意义（*Log*-*rank*, PFS/OS: *P* < 0.001）。上述研究表明：基线不低于10 mg/d泼尼松当量的激素暴露可降低PD-1/PD-L1单抗的疗效获益，显著缩短NSCLC患者的PFS及OS。而深入的分析提示：PD-1/PD-L1单抗治疗前30 d激素暴露也降低了NSCLC患者的疗效获益，但基线GC用药的影响相对更明显。

近期的另一项研究^[26]^纳入了欧洲6个中心（法国5个，荷兰1个）的1, 025例晚期NSCLC患者。所有研究对象均接受PD-1/PD-L1单抗单药或联合CTLA-4单抗治疗。结果显示：141例患者在启动ICIs治疗时曾接受糖皮质激素治疗，GC治疗的适应证为处理脑转移或其他。在多因素分析中，对脑转移情况、体能状况、癌转移器官个数、吸烟史及激素使用情况等因素进行调整后，结果表明：ICIs治疗初持续的激素暴露显著降低了晚期NSCLC患者的PFS及OS（PFS: HR=1.31, *P*=0.01; OS: HR=1.46, *P*=0.001）。该项研究的优点是样本量较大，不足之处在于使用任意剂量GC的患者均被纳入分析，激素的剂量及使用指征等信息并未详细描述。

此外，Scott等^[27]^的小样本研究同样报道：早期持续激素暴露显著降低了nivolumab单药治疗的NSCLC患者的OS。该研究纳入了210例nivolumab单药治疗的NSCLC患者，共66例患者于nivolumab治疗期间接受过GC治疗，剂量从10 mg/d-180 mg/d不等，平均35 mg/d泼尼松当量，激素适应证包括脑转移/放射性损伤（27%）、呼吸道症状（21%）、疲劳等全身症状（18%）、irAEs的管理（17%）及其他指征（17%）。研究将启动nivolumab治疗30 d内，接受不低于10 mg/d泼尼松当量GC治疗定义为早期使用激素，共计25例。分析显示：早期激素暴露组的中位nivolumab治疗周期数较非早期激素暴露组减少（2个周期*vs* 5个周期，*P*=0.002），且中位OS显著缩短（4.3个月*vs* 11个月，*Log*-*rank*，*P*=0.017）。对nivolumab治疗首日GC用药，即基线GC暴露的亚组（*n*=12）进一步分析，其OS较无基线暴露者，亦明显降低（*P*=0.02）。另外，*Cox*回归分析表明：早期激素暴露组的死亡风险较非早期激素暴露组显著增高（HR=2.30, 95%CI: 1.27-4.16, *P*=0.006）。

上述研究均表明：在ICIs治疗早期，持续的GC暴露将显著降低NSCLC患者的疗效。不过近期也有文献报道了相反的结果。Biagio等^[28]^的研究纳入了650例在丹娜法伯癌症中心（Dana-Farber Cancer Institute）接受PD-1/PD-L1单抗单药或联合CTLA-4单抗治疗的晚期NSCLC患者，共93例在ICIs治疗24 h内接受口服或静脉GC治疗（≥10 mg/d泼尼松当量）。根据使用原因，研究者进一步将GC暴露患者分为癌症相关症状治疗组和非癌症相关激素使用组。其中癌症相关症状治疗组66例，激素使用原因包括症状性脑转移及癌症相关气促、骨痛、厌食等。非癌症相关激素使用组27例，激素使用原因包括既往化放疗导致的肺炎、慢性阻塞性肺疾病管理、自身免疫性疾病等。另外557例为无GC用药组。结果显示：在不考虑GC适应证时，总激素治疗患者（*n*=93）的ORR显著低于无激素治疗组（10.8% *vs* 19.7%, *P*=0.04），PFS及OS也均显著缩短（中位PFS：2.0个月*vs* 3.4个月，HR=1.36，*P*=0.01；中位OS：4.9个月*vs* 11.2个月；HR=1.68，*P* < 0.01）。不过，对激素组患者分组分析显示：癌症相关症状治疗组对比无激素治疗组，PFS及OS均显著缩短（PFS: HR=1.87, *P* < 0.001; OS: HR=2.38, *P*=0.001）；而非癌症相关激素使用组对比无激素治疗组，PFS及OS则无显著差异（PFS: HR=0.77, *P*=0.24; OS: HR=0.93, *P*=0.77）。多因素分析的结果同样支持：因癌症相关症状而使用不低于10 mg泼尼松的患者较无激素组患者死亡风险更高（HR=1.60, *P*=0.02）；而非癌症相关激素使用患者，GC的应用并未显著降低其ICIs疗效获益（PFS: HR=0.61, *P* < 0.001; OS: HR=0.32, *P* < 0.001）。最后研究者认为：接受基线GC治疗的患者，其较差的预后主要归因于自身合并其他更多预后不良的因素；若激素使用适应证非癌症相关，则并不显著影响NSCLC患者ICIs的疗效获益。

综上所述，对于ICIs治疗早期持续激素暴露是否对ICIs疗效存在负面影响，目前存在一定争议。尽管最新的Biagio等^[28]^的研究指出：持续基线GC用药本身可能并不显著影响患者ICIs的疗效获益，但此前多项研究却均表明：ICIs治疗早期持续GC暴露显著降低了NSCLC患者的ICIs疗效，而且，其中两项研究在多因素分析时，也充分考虑了脑转移、体能状况评分等预后不良因素。因此，在非必要情况下，ICIs治疗早期仍需慎重考虑持续使用超生理剂量的GC。当然，上述研究均为回顾性分析，存在一定的偏倚，而且部分研究样本量较小，更确切可靠的研究结论待进一步的前瞻性研究及基础研究补充。

### 2.2 化疗前GC预处理对ICIs疗效的影响

前面的分析显示：在ICIs治疗早期，持续超生理剂量的GC暴露可能会损害NSCLC患者ICIs的疗效获益。不过，多项Ⅱ期-Ⅲ期随机临床试验发现，超生理剂量的激素短期用于化疗前预处理，并未显著影响ICIs的疗效。Keynote021是一项随机、对照、开放标签的Ⅱ期临床试验^[15]^，旨在评估pembrolizumab联合含铂双药化疗对比单纯化疗的差异。研究入组了123例无突变晚期非鳞NSCLC患者，随机进行培美曲塞+卡铂化疗（*n*=63），或者pembrolizumab联合培美曲塞+卡铂治疗（*n*=60）。两组均使用GC进行化疗前预处理，结果显示：化疗联合免疫治疗组ORR更高（55% *vs* 29%, *P*=0.001, 6），PFS更长（中位PFS：13.0个月*vs* 8.9个月；HR=0.53，*P*=0.010），疗效获益更佳。此后，多项Ⅲ期临床试验^[34-36]^研究结果同样证实，尽管化疗预处理使用了激素，免疫联合化疗对比单纯化疗治疗晚期NSCLC，均显著改善了PFS和OS，侧面反映超生理剂量的短期激素用于化疗前预处理，可能并不会降低ICIs的疗效获益。综上，在ICIs治疗早期，短暂的超生理剂量的激素用药可能是允许的，至少，激素短期用于化疗前预处理是安全的。不过，仍有一些问题需要解答：①免疫联合化疗的协同作用是否对GC的负效应存在“抵消”作用？②免疫联合化疗前进行或不进行激素预处理对最终疗效是否存在差异？后续可对相关问题进行深入研究。

### 2.3 GC的应用剂量对疗效的影响

Kathryn等^[25]^的研究对基线GC剂量与PD-1/PD-L1单抗疗效的关系进行了探讨，结果显示：基线GC用药（≥10 mg/d泼尼松当量）不仅显著降低NSCLC患者的疗效获益，且其影响存在一定的“量效”关系。研究按GC治疗剂量将患者进行分组，其中58例患者接受不低于20 mg/d泼尼松当量激素，32例接受10 mg/d-19 mg/d泼尼松当量激素，剩余548例为无基线GC用药患者。结果显示：不低于20 mg激素暴露组的ORR为5%，10 mg-19 mg激素暴露组为10%，而无激素暴露组ORR最高，达18%。三者的PFS、OS也存在同上对比关系，不低于20 mg激素暴露组的PFS、OS均最低，三组差异有统计学意义（*Log*-*rank*, OS/PFS: *P* < 0.001）。该研究提示，对于PD-1/PD-L1单抗治疗的患者，基线GC用药的剂量越高，疗效减低可能会越明显。不过，最近Biagio等^[28]^的研究认为：GC治疗的患者预后较差，主要归因于自身合并其他预后不良的因素，而并不一定是激素对ICIs疗效的抑制作用所致。而对于预后较差的患者，其缓解癌症相关症状所依赖的激素剂量亦可能更高，因而也可能表现出一定的“量效”关系。当然，目前相关研究证据不足，GC应用剂量与ICIs疗效关系的迷雾期待更多高质量的研究去拨开。

## 3 GC应用于irAEs管理

### 3.1 ICIs治疗期间irAEs的发生现状及其管理

irAEs是ICIs治疗过程中特殊的不良反应，可累及全身的许多器官系统，包括皮肤、胃肠道、肝脏、内分泌系统及其肺脏等。irAEs谱在CTLA-4与PD-1/PD-L1抑制剂间无明显差异，但后者的治疗不良反应相对较少、较轻^[37]^。虽然irAEs的总发生率可能较高，但大多数为轻度的irAEs。有*meta*分析^[38]^显示，进行CTLA-4抑制剂（3 mg/kg或10 mg/kg）治疗的患者，其任何级别的irAEs总发生率为72%，而中-重度（3级及以上）irAEs发生率为24%。对于PD-1/PD-L1抑制剂，其中-重度irAEs发生率则尚不足20%^[14, 31]^。目前，针对中-重度irAEs，往往需给予一定的全身免疫抑制治疗，而GC为最主要的干预药物^[14, 39]^。

### 3.2 GC用于irAEs管理的安全性

关于GC用于irAEs管理的安全性问题，目前有部分研究进行了初步探索。在这些研究^[29-33, 40]^中，仅一篇文献为前瞻性研究。所应用的ICIs包括CTLA-4、PD-1/PD-L1抑制剂。治疗瘤种虽涵盖黑色素瘤、NSCLC、肾细胞癌、头颈部肿瘤等，但以黑色素瘤为主。GC应用剂量及应用时间信息大多未详细描述。研究终点有OS、PFS、ORR、治疗失败时间（time to treatment failure, TTF）及缓解持续时间（duration of response, DoR）。具体数据汇总见表 1。

**1:** GC用于irAEs管理对ICIs的疗效影响分析 Analysis of the steroid effect on ICIs efficacy for the management of irAEs

Author (year)	Study design	Tumors	ICIs	With/no steroids given	Steroid effect on ICIs efficacy	Detailed description
Horvat (2015)^[29]^	Retrospective	Melanoma	Ipilimumab	80/182	No	No difference in OS (*P*=0.97) or TTF (*P*=0.07) between patients who did and did not receive systemic steroids for irAEs
Johnson (2015)^[30]^	Retrospective	Melanoma	Ipilimumab	12 /21	No	No difference in OS (*P*=0.31) between patients who did and did not receive systemic steroids for irAEs
Weber (2017)^[31]^	Retrospective (4 trials)	Melanoma	Nivolumab	114/462	No	No difference in ORR (*P*=0.74) between patients who did and did not receive systemic steroids for irAEs
Downey (2007)^[32]^	Joint analysis of 2 prospective trials	Melanoma	Ipilimumab ±peptide vaccines	Responders: 12/11 Nonresponders: 26/90	No	No DoR difference in responders who did and did not receive steroids (*P*=0.23); No OS difference in nonresponders who did and did not receive steroids (*P*=0.99)
Shafqat (2018)^[33]^	Retrospective	NSCLC (49) Melanoma (49) Renal cell carcinoma (30) Head and neck cancer (19) Other (16)	Nivolumab Pembrolizumab Atezolizumab	21/136	Improved PFS in all patients and no effect in responders	Patients who received corticosteroid treatment for their irAEs have improved PFS (HR=0.38, *P*=0.03); No PFS difference in responders who did and did not receive steroids (*P*=0.23)
Faje (2018)^[40]^	Retrospective	Melanoma	Ipilimumab	64/217	Improved OS	Patients who developed hypophysitis (receive steroids) had improved OS (median OS: 28.2 months versus 9.5 months; HR=0.53, *P* < 0.01) compared with those who did not have hypophysitis
NSCLC: non-small cell lung cancer; OS: overall survival; PFS: progression-free survival; HR: hazard ratio; ORR: overall response rate; TTF: time to treatment failure; DoR: duration of response; irAEs: immune-related adverse events.						

上述研究均表明：治疗剂量的GC用于irAEs管理对ICIs抗肿瘤疗效并无显著的负面影响，从机制上讲，这种现象可能是合理的。正常生理过程中，CTLA-4与PD-1/PD-L1为一类负向调节机体免疫应答的分子，主要参与维持机体免疫耐受，避免过强的免疫应答损伤自身组织，维持机体的免疫平衡。但ICIs的介入解除了免疫检查点对机体免疫系统的“刹车”效应，使机体的免疫平衡被打破。irAEs的出现不仅标志着机体免疫系统的激活，更意味着过强的免疫系统开始攻击正常组织，此时应用治疗剂量的GC，可调节机体免疫处于一个合适的强度，而并不显著降低ICIs的疗效。另外，有基础研究^[22]^显示：在经地塞米松处理的T细胞群中，未致敏的原始T细胞的增殖显著被抑制，但经抗原激活后的记忆T细胞亚群对地塞米松的增殖抑制作用并不敏感，提示在抗肿瘤免疫建立后，T细胞系统可能对激素的负性效应具有较好的适应能力，这在一定程度上也支持当前的研究结论。不过，目前评估GC用于irAEs管理安全性的研究中，所有的GC干预对象均为irAEs患者，而多项研究^[33, 41, 42]^表明，发生irAEs与更佳的抗肿瘤获益有关，预示着GC对ICIs治疗的负面影响也可能伴随着irAE的疗效获益被“掩盖”。

Faje等^[40]^的研究对比了不同剂量GC治疗ipilimumab诱导的垂体炎对患者预后的影响。研究确认了98例接受ipilimumab治疗并被诊断为垂体炎的黑色素瘤患者，最终64例ipilimumab单药治疗患者纳入分析，包括激素低剂量组14例，高剂量组50例。低剂量组GC剂量≤7.5 mg/d泼尼松当量，中位GC剂量5.5 mg，高剂量组GC剂量均 > 7.5 mg/d泼尼松当量，中位GC剂量22.4 mg。结果表明，高剂量组的中位OS、PFS及TTF分别为23.3个月、4.9个月及11.4个月，而低剂量组中位PFS为35.0个月，且尚未达到其中位OS及TTF。低剂量组的OS（HR=0.24, *P*=0.002）、PFS（HR=0.36, *P*=0.007）及TTF（HR=0.28, *P*=0.001）均更优。进一步对比垂体炎患者（*n*=64）和同期非垂体炎患者（*n*=217）的生存情况，发现垂体炎患者的OS更长（中位OS：28.2个月*vs* 9.7个月；HR=0.53，*P*=0.000, 3），1年生存率及2年生存率均更高（83% *vs* 45%; 59% *vs* 35%）。研究提示：用于irAEs管理时，高剂量GC对ICIs疗效仍存在潜在的负面影响。不过，irAEs患者通常具有更佳的ICIs疗效，体内的免疫活性相对更强，因而尽管接受GC干预治疗，其ICIs疗效较无irAEs患者仍可能更优。

综上，我们认为：治疗剂量的GC用于irAEs的管理整体上是安全的。形成这种安全性的原因可能有两方面，一方面可能由于发生irAEs时，机体抗肿瘤免疫已不同程度建立，此时的T细胞系统对GC的免疫抑制作用具有良好适应能力；另一方面，irAEs的出现通常标志着机体更强的免疫活性，治疗剂量的GC可有效调节机体免疫处于合理的强度，而并不会显著影响ICIs的抗肿瘤疗效。不过，高剂量的GC对ICIs治疗仍可能是有害的，故在治疗irAEs时，需避免使用或慎用高剂量激素。另外，当前研究中GC使用剂量和使用时间等信息并不充分明确，而临床上irAEs类型具有多样性，其严重程度也不相同，存在明显的个体差异，因而用于irAEs管理的GC治疗剂量存在一定的异质性。对于如何确定各irAEs激素治疗剂量的安全范围以及irAEs患者是否存在明确的GC阈治疗剂量等问题，需要后续研究跟进探索。

## 4 GC对不同ICIs疗效影响是否有差异？

目前多项Ⅱ期-Ⅲ期随机临床试验^[15, 34-36]^显示：GC用于联合治疗化疗前预处理时，对于联合各类PD-1/PD-L1单抗治疗的NSCLC患者，其ICIs疗效均未受到显著影响，提示在化疗前预处理时，激素对各类PD-1/PD-L1单抗的疗效影响可能并无显著的差异。对于管理irAEs，基于多项回顾性分析^[29-33]^的结果：不管是PD-1/PD-L1单抗还是CTLA-4单抗，激素对其疗效也均无显著的负面影响。不过，上述两种情况均为阴性结果，即激素对ICIs疗效均无显著影响，目前暂不明确激素是否产生了确切有效的影响效应，因此，不能绝对认为GC对不同ICIs的影响并无差异。对于激素用于肿瘤相关并发症及其他适应证时，其对不同ICIs的影响是否存在差异，目前缺乏单纯对比的证据。

## 5 总结与展望

本综述的结果显示：对于ICIs治疗早期，持续性的GC暴露（不低于10 mg/d泼尼松当量）是否会影响ICIs的疗效，目前还存在一定争议。但是，超生理剂量激素短暂用于免疫联合化疗的预处理，则并不显著影响ICIs的疗效获益。因此，在非必要情况下，ICIs治疗早期应谨慎持续使用超生理剂量的糖皮质激素。对于irAEs的管理，治疗剂量的GC用药总体上是安全的，不过临床上仍需合理评估GC的使用指征及剂量强度，尽量减少高剂量使用。上述结论是初步的，目前仍有许多问题尚待解决。首先，当前评估GC用于ICIs治疗早期或irAEs管理期的研究存在瘤种单一、样本量小等局限。不同肿瘤的生物学行为、疗效反应等存在异质性，研究结论在不同瘤种间是否普适，值得进一步探讨。再者，关于irAEs管理时GC用药是否存在剂量上限及最小阈值剂量等问题，仍需更多研究探索分析。总之，有关GC对ICIs疗效影响的探索，目前仅有一些粗浅的研究结论，未来亟需对这个领域开展全方位、系统性的前瞻性研究。

## References

[1] Nadal E, Massuti B, Domine M (2019). Immunotherapy with checkpoint inhibitors in non-small cell lung cancer: insights from long-term survivors. Cancer Immunol Immunother.

[b2] 2Payandeh Z, Yarahmadi M, Nariman-Saleh-Fam Z, *et al.* Immune therapy of melanoma: Overview of therapeutic vaccines. J Cell Physiol, 2019. [Epub ahead of print] doi: 10.1002/jcp.28181

[3] Motzer RJ, Escudier B, McDermott DF (2015). Nivolumab versus everolimus in advanced renal-cell carcinoma. N Engl J Med.

[4] Armand P, Shipp MA, Ribrag V (2016). Programmed death-1 blockade with pembrolizumab in patients with classical Hodgkin lymphoma after brentuximab vedotin failure. J Clin Oncol.

[5] Brahmer J, Reckamp KL, Baas P (2015). Nivolumab versus docetaxel in advanced squamous-cell non-small-cell lung cancer. N Engl J Med.

[6] Borghaei H, Paz-Ares L, Horn L (2015). Nivolumab versus docetaxel in advanced nonsquamous non-small-cell lung cancer. N Engl J Med.

[7] Herbst RS, Baas P, Kim DW (2016). Pembrolizumab versus docetaxel for previously treated, PD-L1-positive, advanced non-small-cell lung cancer (KEYNOTE-010): a randomised controlled trial. Lancet.

[8] Rittmeyer A, Barlesi F, Waterkamp D (2017). Atezolizumab versus docetaxel in patients with previously treated non-small-cell lung cancer (OAK): a phase 3, open-label, multicentre randomised controlled trial. Lancet.

[9] Reck M, Rodriguez-Abreu D, Robinson AG (2016). Pembrolizumab versus chemotherapy for PD-L1-positive non-small-cell lung cancer. N Engl J Med.

[10] Oppong E, Cato AC (2015). Effects of glucocorticoids in the immune system. Adv Exp Med Biol.

[11] Ryken TC, McDermott M, Robinson PD (2010). The role of steroids in the management of brain metastases: a systematic review and evidence-based clinical practice guideline. J Neurooncol.

[12] Paulsen O, Klepstad P, Rosland JH (2014). Efficacy of methylprednisolone on pain, fatigue, and appetite loss in patients with advanced cancer using opioids: a randomized, placebo-controlled, double-blind trial. J Clin Oncol.

[13] Lin RJ, Adelman RD, Mehta SS (2012). Dyspnea in palliative care: expanding the role of corticosteroids. J Palliat Med.

[14] Puzanov I, Diab A, Abdallah K (2017). Managing toxicities associated with immune checkpoint inhibitors: consensus recommendations from the Society for Immunotherapy of Cancer (SITC) Toxicity Management Working Group. J Immunother Cancer.

[15] Langer CJ, Gadgeel SM, Borghaei H (2016). Carboplatin and pemetrexed with or without pembrolizumab for advanced, non-squamous non-small-cell lung cancer: a randomised, phase 2 cohort of the open-label KEYNOTE-021 study. Lancet Oncol.

[16] Elias R, Karantanos T, Sira E (2017). Immunotherapy comes of age: Immune aging & checkpoint inhibitors. J Geriatr Oncol.

[17] Kim HD, Park SH (2019). Immunological and clinical implications of immune checkpoint blockade in human cancer. Arch Pharm Res.

[18] Freeman GJ, Long AJ, Iwai Y (2000). Engagement of the PD-1 immunoinhibitory receptor by a novel B7 family member leads to negative regulation of lymphocyte activation. J Exp Med.

[19] Francisco LM, Salinas VH, Brown KE (2009). PD-L1 regulates the development, maintenance, and function of induced regulatory T cells. J Exp Med.

[20] Dong H, Strome SE, Salomao DR (2002). Tumor-associated B7-H1 promotes T-cell apoptosis: a potential mechanism of immune evasion. Nat Med.

[21] Bianchi M, Meng C, Ivashkiv LB (2000). Inhibition of IL-2-induced Jak-STAT signaling by glucocorticoids. Proc Natl Acad Sci U S A.

[22] Giles AJ, Hutchinson MND, Sonnemann HM (2018). Dexamethasone-induced immunosuppression: mechanisms and implications for immunotherapy. J Immunother Cancer.

[23] Chen X, Murakami T, Oppenheim JJ (2004). Differential response of murine CD4^+^CD25^+^ and CD4^+^CD25^-^ T cells to dexamethasone-induced cell death. Eur J Immunol.

[24] Chen X, Oppenheim JJ, Winkler-Pickett RT (2006). Glucocorticoid amplifies IL-2-dependent expansion of functional FoxP3(+)CD4(+)CD25(+) T regulatory cells *in vivo* and enhances their capacity to suppress EAE. Eur J Immunol.

[25] Arbour KC, Mezquita L, Long N (2018). Impact of baseline steroids on efficacy of programmed cell death-1 and programmed death-ligand 1 blockade in patients with non-small-cell lung cancer. J Clin Oncol.

[26] Hendriks LEL, Henon C, Auclin E (2019). Outcome of patients with non-small cell lung cancer and brain metastases treated with checkpoint inhibitors. J Thorac Oncol.

[27] Scott SC, Pennell NA (2018). Early use of systemic corticosteroids in patients with advanced NSCLC treated with nivolumab. J Thorac Oncol.

[28] Ricciuti B, Dahlberg SE, Adeni A (2019). Immune checkpoint inhibitor outcomes for patients with non-small-cell lung cancer receiving baseline corticosteroids for palliative versus nonpalliative indications. J Clin Oncol.

[29] Horvat TZ, Adel NG, Dang TO (2015). Immune-related adverse events, need for systemic immunosuppression, and effects on survival and time to treatment failure in patients with melanoma treated with ipilimumab at memorial Sloan Kettering Cancer Center. J Clin Oncol.

[30] Johnson DB, Friedman DL, Berry E (2015). Survivorship in immune therapy: Assessing chronic immune toxicities, health outcomes, and functional status among long-term ipilimumab survivors at a single referral center. Cancer Immunol Res.

[31] Weber JS, Hodi FS, Wolchok JD (2017). Safety profile of nivolumab monotherapy: A pooled analysis of patients with advanced melanoma. J Clin Oncol.

[32] Downey SG, Klapper JA, Smith FO (2007). Prognostic factors related to clinical response in patients with metastatic melanoma treated by CTL-associated antigen-4 blockade. Clin Cancer Res.

[33] Shafqat H, Gourdin T, Sion A (2018). Immune-related adverse events are linked with improved progression-free survival in patients receiving anti-PD-1/PD-L1 therapy. Semin Oncol.

[34] Socinski MA, Jotte RM, Cappuzzo F (2018). Atezolizumab for first-line treatment of metastatic nonsquamous NSCLC. N Engl J Med.

[35] Paz-Ares L, Luft A, Vicente D (2018). Pembrolizumab plus chemotherapy for squamous non-small-cell lung cancer. N Engl J Med.

[36] Gandhi L, Rodriguez-Abreu D, Gadgeel S (2018). Pembrolizumab plus chemotherapy in metastatic non-small-cell lung cancer. N Engl J Med.

[37] Cousin S, Italiano A (2016). Molecular pathways: Immune checkpoint antibodies and their toxicities. Clin Cancer Res.

[38] Bertrand A, Kostine M, Barnetche T (2015). Immune related adverse events associated with anti-CTLA-4 antibodies: systematic review and *meta*-analysis. BMC Med.

[39] Champiat S, Lambotte O, Barreau E (2016). Management of immune checkpoint blockade dysimmune toxicities: a collaborative position paper. Ann Oncol.

[40] Faje AT, Lawrence D, Flaherty K (2018). High-dose glucocorticoids for the treatment of ipilimumab-induced hypophysitis is associated with reduced survival in patients with melanoma. Cancer.

[41] Freeman-Keller M, Kim Y, Cronin H (2016). Nivolumab in resected and unresectable metastatic melanoma: characteristics of immune-related adverse events and association with outcomes. Clin Cancer Res.

[42] Fujii T, Colen RR, Bilen MA (2018). Incidence of immune-related adverse events and its association with treatment outcomes: the MD Anderson Cancer Center experience. Invest New Drugs.

